# Interleukin-1 beta guides the migration of cortical neurons

**DOI:** 10.1186/1742-2094-11-114

**Published:** 2014-06-21

**Authors:** Lei Ma, Xiao-wei Li, Shi-jun Zhang, Feng Yang, Ge-min Zhu, Xiao-bing Yuan, Wen Jiang

**Affiliations:** 1 Department of Neurology, Xijing Hospital, Fourth Military Medical University, Xi’an 710032, China; 2 Institute of Neuroscience and Key State Laboratory of Neurobiology, Shanghai Institute for Biological Sciences, Chinese Academy of Sciences, Shanghai 200031, China

**Keywords:** IL-1beta, Neuronal migration, *in utero* electroporation, Brain development

## Abstract

**Background:**

Proinflammatory cytokine interleukin-1beta (IL-1β) is expressed at high levels in the developing brain and declines to low constitutive levels in the adult. However, the pathophysiological function of IL-1β during brain development remains elusive. In this study, we investigated the role of IL-1β in neuronal migration.

**Methods:**

The Boyden transwell assay was used to examine the effects of IL-1β on the migration of dissociated primary cortical neurons. To determine the role of IL-1β in neuron leading process pathfinding, we employed a growth cone turning assay. *In utero* electroporation combined with RNAi technology was used to examine the neuronal migration *in vivo* during brain development in Sprague–Dawley rats.

**Results:**

IL-1β at concentrations ranging from 0.1 to 10 ng/mL in the lower chamber of a transwell induced a significant increase in the number of migrating neurons in a dose-dependent manner. When IL-1β was simultaneously put in both the upper and lower chambers to eliminate the gradient, no significant differences in cell migration were observed. IL-1 receptor antagonist IL-1RA dose-dependently blocked the attractive effect of IL-1β on neuronal migration. Microscopic gradients of IL-1β were created near the growth cones of isolated neurons by repetitive pulsatile application of picoliters of a IL-1β-containing solution with a micropipette. We found that growth cones exhibited a clear bias toward the source of IL-1β at the end of a one hour period in the IL-1β gradient. No significant difference was observed in the rate of neurite extension between IL-1β and controls. We electroporated specific siRNA constructs against IL-1R1 mRNA into cortical progenitors at embryonic day 16 and examined the position and distribution of transfected cells in the somatosensory cortex at postnatal day 5. We found that neurons transfected with IL-1R1-siRNA displayed a severe retardation in radial migration, with about 83% of total cells unable to arrive at the upper cortical layers.

**Conclusions:**

Our study suggests an essential contribution of IL-1β to neuronal migration during brain development, which provides a basis to understand the physiological roles of IL-1β in the developing brain and could have significant implications for the prevention of some neurodevelopment disorders due to abnormal neuronal migration.

## Background

Interleukin-1beta (IL-1β), one of the most widely studied proinflammatory cytokines, is expressed at high levels in the brain during prenatal and postnatal development and declines to low constitutive levels in the adult [1,2], suggesting its important role in brain development. Mounting evidence shows that exposure to environmental insults as well as other adverse events during brain development, such as brain injury, infection and stress, can cause some neurodevelopmental disorders, such as cortical dysplasia [3]. IL-1β can be significantly activated in the central nervous system (CNS) by these injuries [4]. Elevated levels of IL-1β in the circulation shortly after preterm birth are also associated with increased risk of neurodevelopmental disorders [5]. This evidence indicates that dysfunction of IL-1β signaling might be involved in the pathogenesis of some neurodevelopmental disorders.

Previous studies showed that IL-1β can regulate the migration of many types of cell, such as smooth muscle cells [6], tumor cells [7], airway epithelial cells [8], neutrophil [9] and astrocyte progenitors [10]. Our pilot experiment revealed that IL-1β was able to attract the migration of cultured cortical neurons. Neuronal migration is an important feature of the cortical development stage, during which postmitotic neurons migrate away from the ventricular zone along radial glial fibers and toward the surface of the cortical plate. Once disturbance of normal migration occurs, neurons may accumulate in unusual areas (heterotopias), resulting in either focal neuronal heterotopias (nodular heterotopias) or diffuse band heterotopias in the white matter (pachygyrias/lissencephalies, double cortex syndrome) [11]. Thus, studies of whether IL-1β regulates neuronal migration are important to further our understanding of brain development and the pathogenesis of some neurodevelopmental disorders.

In this study, we first investigated the effect of IL-1β on neuronal migration *in vitro* by using a transwell migration assay and growth cone turning assay. Then we applied RNAi technology combined with *in utero* electroporation to demonstrate that IL-1β can guide the radial migration during development of the rat neocortex.

## Methods

### Animals

All pregnant Sprague–Dawley rats used in the present study were provided by the Fourth Military Medical University Animal Center (Xi’an, China) and SLAC Laboratory Animal Co. Ltd (Shanghai, China). The rats were housed under controlled temperature and light conditions (12-hour light/dark cycle with lights on at 8:00 AM), with *ad libitum* access to food and water. The day at which a vaginal plug was detected was designated embryonic day 0 (E0). All experimental procedures involving rats were in strict accordance with the guidelines established by the US National Institutes of Health (NIH) and were approved by the Fourth Military Medical University Animal Care Committee. Procedures for *in utero* electroporation and growth cone turning assay were approved by the Animal Care and Administration Committee of the Institute of Neuroscience, Shanghai Institute for Biological Sciences, Chinese Academy of Sciences.

### Cell culture and immunocytochemistry

Primary culture of cerebral cortical neurons was performed in accordance with previous methods [12]. Briefly, cortical tissues from E16 rats were dissected and digested by 0.125% trypsin in phosphate-buffered saline (PBS), and dissociated neurons were plated into 35 mm dishes coated with 100 mg/mL poly-D-lysine. In transfection experiments, cells were transfected with 3 μg of different plasmids by using the Amaxa Nucleofector kit (Amaxa GmbH, Cologne, Germany) following the protocol provided by the manufacturer.

For the detection of IL-1R1 expression *in vitro*, cultured cortical neurons at five day *in vitro* (DIV) were fixed with 4% paraformaldehyde. Then, double immunofluorescence staining with anti-IL-1R1 (polyclonal antibody, Abcam, Cambridge, UK, 1:100) and anti-Tuj1 (polyclonal antibody, Millipore, Bilerica, MA, USA, 1:1000) was performed according to the protocol described previously [13]. The specificity of immunolabeling was verified by controls in which the primary antibody was omitted.

### Cell migration assay

Migration of dissociated primary cortical neurons was assayed by using a Boyden transwell system (8 μm pore size; Corning Costar, NY, USA) as described previously [14]. Before seeding, both sides of the transwell were coated overnight with poly-D-lysine (30 μg/mL, Sigma-Aldrich, Saint Louis, MO, USA). Serum-free medium, 250 μl, (neurobasal medium, 2% B27, Gibco, Carlsbad, CA, USA) containing dissociated cells (2 × 10^5^ cells per well) was added to the upper insert of a chamber with or without any other reagents. In the bottom chamber, 750 μl of serum-free medium (Neurobasal medium, 2% B27, Gibico) with or without any other reagents was added. Reagents used were IL-1β (PeproTech, London, UK, 0.1 ng/ml, 1 ng/ml, 10 ng/ml) and IL-1RA (R & D, Minneapolis, Minn., USA, 1 ng/ml, 10 ng/ml, 100 ng/ml). Twenty hours after seeding, cells were fixed with 4% paraformaldehyde, and cells attached to the upper side of the membranes were thoroughly scraped off. Cells attached to the bottom side of the membranes were stained with coomassie brilliant blue. Cells were counted from five randomly chosen fields (magnification, × 200) for each membrane under the microscope. Each experiment was repeated at least four times.

### Growth-cone turning assay

Cerebellar tissues from Sprague–Dawley rats (Postnal day (P) 0 to P2) were incubated in 0.1% trypsin (Sigma) in PBS for 10 minutes at 37°C, followed by trituration. Dissociated cells were resuspended and plated on coverslips coated with laminin (25 mg/ml; Sigma) and were used for experiments 18 to 24 hours after plating at room temperature (20 to 22°C). Quantitative assay of growth cone turning was performed according to a method described previously [15]. The pipette tip (1 μm opening) containing the chemical was placed 100 μm away from the center of the growth cone of an isolated neuron and at an angle of 45° with respect to the initial direction of neurites (indicated by the last 10 μm segment of the neurite). A standard pressure pulse of 3 psi was applied at a frequency of 2 Hz with durations of 20 ms. The turning angle was defined by the angle between the original direction of neurite extension and a straight line connecting the positions of the growth cone at the onset and the end of the one-hour period. Neurite extension was quantified by measuring the entire trajectory of net neurite growth over the one-hour period. Only those growth cones with net extension >10 μm over the one-hour period were included for analysis of turning angles. Data are presented as mean ± standard error of the mean (SEM). Statistical significance was analyzed by Kolmogorov–Smirnov test.

### Plasmid construction

The IL-1R1-siRNA sequences were designed using an online design tool (http://dharmacon.gelifesciences.com) and cloned into a pSuper vector under the control of H1 promoter. The siRNA sequences are given as follow:

IL-1R1-RNAi1 (IL-1R1-i1): 5′-GCTGTCCTCTTACTCCAAA-3′;

IL-1R1-RNAi2 (IL-1R1-i2): 5′-GGAGACACACTTACCACTT-3′;

Scramble control (Scramble): 5′-CAGTCGCGTTTGCGACTGG-3′.

To construct the overexpression plasmid of the RNAi-resistant human IL-1R1 homologue (h-IL-1R1), full-length human IL-1R1 was amplified by PCR using primers IL-1R1 hFW and IL-1R1 hRW from human muscle cDNA (a gift from Dr. Shi) and subcloned into the AscI/XhoIsite of pCAG-IRES-EGFP. The sequences of the primers used are shown in Table 1. All the re-constructed plasmids were verified by sequencing.

**Table 1:** **Sequences of PCR primers for RT**-**PCR experiments**

**Items**	**Sequence**
IL-1β RTFW	5'-AATGACCTGTTCTTTGAGGCTGAC-3'
IL-1β RTRW	5-'CGAGATGCTGC TGTGAGATTTGAAG-3'
IL-1R1 RTFW	5'-AGAAACTCAACATACTGCCTCA-3'
IL-1R1 RTRW	5'-CAGCCACATTCATCACCATC-3'
β-actin RTFW	5'-CGTTGACATCCGTAAAGAC-3'
β-actin RTRW	5'-TGGAAGGTGGACAGTGAG-3'
IL-1R1 hFW	5'-TTGGCGCGCCATGAAAGTGTTACTCAGACT-3'
IL-1R1 hRW	5'-CCGCTCGAGCTACCCGAGAGGCACGTGAG-3'

### Western blotting

Western blotting was performed as described previously [12,14]. Briefly, primary cultured neurons were lysed in 0.2 mL of lysis buffer (0.1% sodium dodecyl sulfate, 1% Nonidet P-40, 50 mM N-2-hydroxyethylpiperazine-N-2-ethanesulfonic acid (pH 7.4), 2 mM ethylenediaminetetraacetic acid (EDTA), 100 mM NaCl, 5 mM sodium orthovanadate, and 40 M p-nitrophenyl phosphate) with 1% Protease Inhibitor Mixture Set I (Calbiochem, Hofheim, Germany). The total protein concentration of the samples was measured by using the Bio-Rad RC/DC reagent kit (Bio-Rad Laboratories, Hercules, CA, USA). Samples of the protein (50 μg) were loaded per lane on 12% SDS-polyacrylamide gels and transferred to nitrocellulose membranes. The samples were blocked with 5% non-fat milk in Tris-buffered saline with Tween (TBST) for two hours and incubated over night at room temperature with rabbit-derived polyclonal anti-IL-1R1 antibody (Abcam, USA, 1:1000). As a protein loading control, the samples were also incubated with mouse-derived monoclonal anti-α-tubulin (Sigma, USA, 1:3000). After being washed with PBS, the samples were incubated for one hour with goat anti-rabbit immunoglobulin G (IgG) conjugated to horseradish peroxidase (1:3000, Bio-Rad, USA). The density of western blotting bands was measured with the Image Quant 5.2 software (GE Healthcare Life Sciences) and quantified as previously reported [12,14].

### *In utero* electroporation

Plasmids were transfected by using *in utero* electroporation (IUE) in accordance with previous methods [12]. Multiparous Sprague–Dawley rats on E16 were anesthetized with 10% chloral hydrate (3.5 ml/kg) intraperitoneally. For electroporation of two vectors, a mixture of EYFP (6 mg/ml) and siRNA (6 mg/ml) constructs was prepared at a ratio of 1:1. Uteruses were exposed, and then 15 to 20 μg of plasmid mixed with Fast Green (2 mg/ml; Sigma) was injected by trans-uterus pressure microinjection into the lateral ventricle of embryos. Next, electric pulses were generated by a pulse generator (BTX T830) and applied to the cerebral wall at five repeats of 60 V for 50 ms, with an interval of 100 ms. In some experiments, bromodeoxyuridine (BrdU; Sigma) was injected at 100 mg/kg intraperitoneally twice every 30 minutes 24 hours after *in utero* electroporation.

Embryonic brains were directly removed and fixed with 4% paraformaldehyde, and postnatal brains at appropriate ages were removed and fixed in 4% paraformaldehyde after transcardial perfusion. For fluorescence immunostaining, both fetal and postnatal brains were cryopreserved in optimal cutting temperature (OCT) compound (Sakura Finetek, Torrence, CA, USA). Coronal cryostat sections of 30 μm were cut on a freezing microtome and immediately processed for immunostaining by the following three-step free-floating protocol: blocking of nonspecific antigenic sites in 5% bovine serum albumin plus 0.2% Triton X-100, overnight incubation with primary antibodies, and overnight incubation with secondary antibodies. The primary antibodies used were anti-IL-1R1 (polyclonal antibody, Abcam, 1:100), anti-Tuj1 (polyclonal antibody, Millipore, 1:1000), anti-GFP (polyclonal antibody, Invitrogen, Carlsbad, CA, USA, 1:1000), anti-BrdU (monoclonal antibody, Sigma, 1:200) and anti-Nestin (monoclonal antibody, Abcam, 1:500). Fluorescently conjugated monoclonal or polyclonal IgG Alexa 488 or Alexa 633 (Molecular Probes; Eugene, OR, 1:1,000) were used as secondary antibodies. Images were acquired using an F1000 confocal system (Olympus) and were processed using Adobe Photoshop CS. For comparison of neuron distribution, the number of GFP^+^ or BrdU^+^ neurons in each subregion (layers II and III, layers IV--VI, IZ-VZ-WM) was counted to calculate the percentages of neurons in each region.

### Statistical analysis

All results are expressed as mean ± SEM. Unless otherwise indicated, one way analysis of variance (ANOVA) followed by a Bonferroni *post hoc* test was used to assess the statistical difference. Statistical significance was set at *P* <0.05. Data management and statistical analyses were performed using SPSS (v11.0).

## Results

### IL-1R1 is expressed in rat cortical neurons *in vitro* and *in vivo*

**Figure 1:**
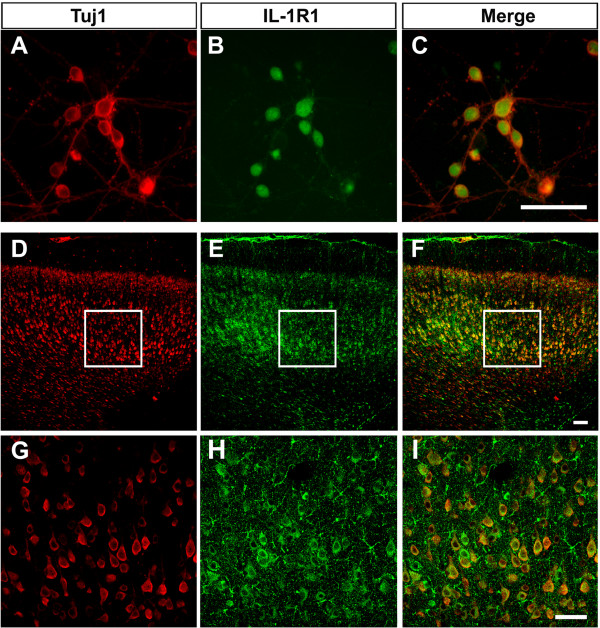
**IL-1R1 is expressed in rat cortical neurons *in vitro *and *in vivo*.** Double-labeling immunofluorescence showed that Tuj1-positive cells colocalized with IL-1R1 in cultured cortical neurons **(A-C)** and in the coronal sections of the rat neocortex at P0 **(D-F)**. The selected regions in **D**-**F** are shown in high magnification in **G**-**I**, respectively. P, postnatal day. Scale bar, 100 μm.

First, we examined whether rat cortical neurons express IL-1R1. Primary cultured neurons from rat cortical tissue were identified by immunostaining with anti-Tuj1 as shown in Figure 1A. We found that there was a strong IL-1R1 immunoreactivity in cortical neurons and that the reactive products were distributed in both the cytoplasm and nuclei of the cells (Figure 1B-C). No immunoreactive products were observed in the methodological control, in which the primary anti-IL-1RI antibody was omitted. To further examine the expression of IL-1R1 in cortical neurons *in vivo*, coronal sections of rat neocortex at P0 were double stained for IL-1R1 and Tuj1. In agreement with the *in vitro* results, Tuj1-positive neurons expressed IL-1R1 (Figure 1D-I). Although the intensity of immunostaining varied from cell to cell, almost all neurons were intensely immunolabeled with IL-1R1.

### IL-1β attracts migration of cortical neurons *in vitro*

**Figure 2:**
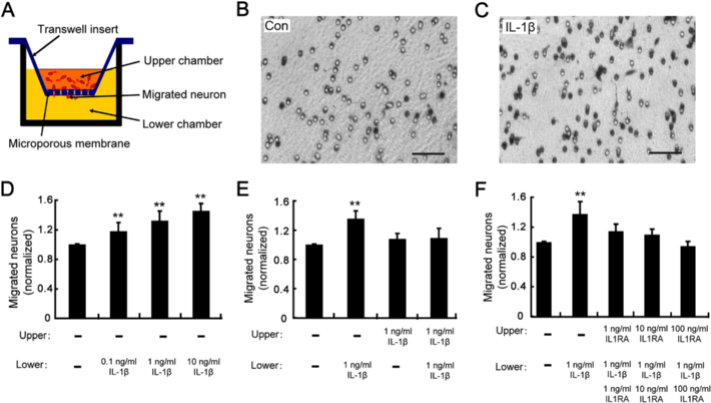
**IL-1β attracts migration of cortical neurons *in vitro*. (A)** Schematic diagram of transwell assay. **(B, C)** Photomicrographs of stained cells that crossed the 8 μm pore after 20 hours of migration in the absence **(B)** or presence **(C)** of IL-1β (1 ng/mL) in the lower chamber. Scale bar, 100 μm. **(D)** Number of migrated neurons normalized to the value of the parallel control when IL-1β was added to the lower chamber at concentrations of 0.1 ng/mL, 1 ng/mL and 10 ng/mL, respectively. **(E)** Number of migrated neurons normalized to the value of the parallel control when IL-1β was added to the upper chamber, lower chamber and both, respectively. **(F)** Number of migrated neurons normalized to the value of the parallel control when IL-1RA at different concentrations was added to the upper and lower chambers before addition of IL-1β in the lower chamber. **P <0.01 versus control group.

Then, we investigated whether IL-1β can regulate neuronal migration *in vitro* by using a transwell migration assay (Figure 2A). Cultured cortical neurons were plated in the upper chamber of transwell plates while IL-1β at concentrations ranging from 0.1 to 10 ng/mL was added to the lower chamber. We observed that IL-1β induced a significant increase in the number of migrated neurons in a dose-dependent manner (Figure 2B-D). To verify whether the results obtained could reflect a change in chemokinetic activity, IL-1β was simultaneously put in both the upper and lower chambers to eliminate the gradient or was put in the upper chamber only. Under these conditions, no significant differences in cell migration were observed (Figure 2E), suggesting that IL-1β is chemotactic rather than chemokinetic for neurons. To test whether the attractive effect of IL-1β on neuronal migration was mediated by its functional receptor IL-1R1, the same experiments were performed in the presence of IL-1RA at different concentrations. We found that IL-1RA dose-dependently blocked the attractive effect of IL-1β on neuronal migration (Figure 2F). Thus, IL-1β is capable of attracting neuronal migration by directly binding its specific receptor IL-1R1.

### IL-1β guides axonal growth cone turning

**Figure 3:**
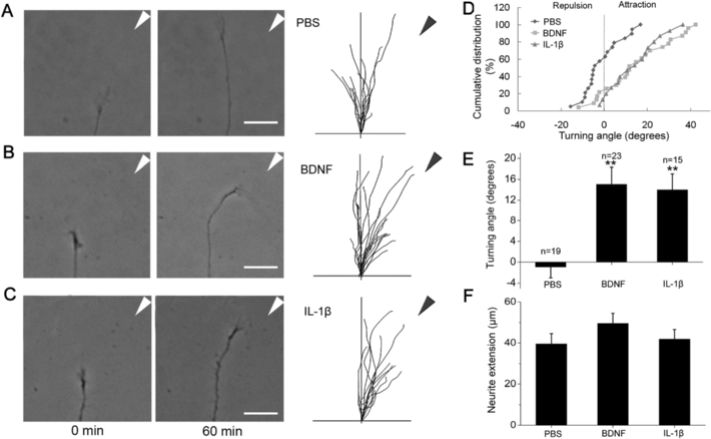
**IL-1β guides axonal growth cone turning. (A-C)** Microscopic images of the growth cone of a cerebellar granule cell at the beginning and the end of a one-hour exposure to a gradient of PBS, BDNF (20 μg/ml in the pipette) or IL-1β (1 μg/ml in the pipette). Superimposed traces (right) depict the trajectory of neurite extension during the one-hour period for all neurons examined. The origin is the center of the growth cone at the onset of the experiment, and the original direction of growth was vertical. The arrow indicates the direction of the gradient. Scale bar, 50 μm. **(D)** The distribution of turning angles. For each experimental condition, the angular positions of all growth cones at the end of a one-hour exposure to a gradient of PBS, BDNF or IL-1β are shown in the cumulative frequency plot. The percentage value refers to the percentage of growth cones with angular position ≤ a given angular value. **(E)** The average turning angle during the one-hour assay for all neurons examined. **(F)** The neurite extension rate during the one-hour assay for all neurons examined. **P <0.01 versus PBS group. BDNF, brain-derived neurotrophic factor.

During neocortex development, migrating neurons possess a short leading process ending in a growth cone, which can sense different types of extracellular guidance cues and thereby contributes to the control of migration direction [16]. To determine the role of IL-1β in leading process pathfinding, we employed a growth cone turning assay. Cerebellar granule cells were used as a model to perform this assay because they constitute a large, homogeneous and easily-cultured population of cells, which maintains migratory behavior, a characteristic that makes them very convenient for *in vitro* assays [17]. Microscopic gradients of IL-1β were created near the growth cones of isolated neurons by repetitive pulsatile application of picoliters of IL-1β-containing solution with a micropipette. The tip of the micropipette was positioned at a distance of 100 μm from the growth cone and at a 45°angle with respect to the direction of neurite extension. The direction and total length of neurite extension were measured one hour after the onset of gradient. When the pipette concentration of IL-1β was 1 μg/ml, we consistently observed an apparent chemotropic turning response of the growth cone toward the pipette, as illustrated in Figure 3. The growth cone behavior of a population of neurons is shown by superimposing traces of individual trajectories of neurite extension over the one-hour period for a random sample of neurons. As depicted in Figure 3C, growth cones exhibited a clear bias toward the source of IL-1β at the end of a one-hour period in the IL-1β gradient. Given that brain-derived neurotrophic factor (BDNF) has been shown to guide neuronal migration during early development [18], we chose it as a positive control. We found that BDNF caused marked attractive growth cone turning whereas PBS had no significant effect on growth cone turning (Figure 3A and B). Statistical analysis of the turning angle further confirmed that IL-1β as well as BDNF is an attractive guidance molecule for the growth cone (Figure 3D and E). No significant difference was observed in the rate of neurite extension among IL-1β, BDNF and PBS groups (Figure 3F).

Recent studies have shown that when the pipette tip was positioned at a distance of 100 μm from the center of the growth cone, the average concentration of the chemical at the growth cone is approximately 10^3^-fold lower than that in the pipette [19]. This suggests that the effective average concentration of IL-1β at the growth cone is about 1 ng/ml, which is similar to that found to promote neuronal migration as above mentioned.

### IL-1R1 RNAi impairs radial migration of cortical neurons

**Figure 4:**
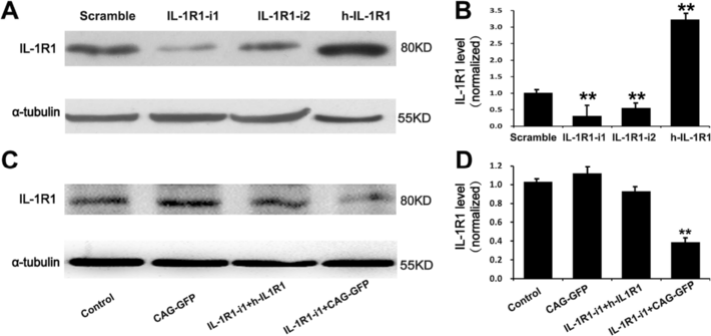
**Down-regulation and up-regulation of IL-1R1 by specific siRNAs and h-IL-1R1, respectively. (A)** Representative western blots showing the down-regulation and up-regulation of IL-1R1 by specific siRNAs or human IL-1R1 homologue (h-IL-1R1) in cultured cortical neurons, respectively. **(B)** Histograms represent the average expression of IL-1R1, normalized to the value of the parallel control. Quantitative data are shown as mean ± SEM from three independent experiments. **P <0.01, versus scramble group. **(C)** Representative western blots showing the rescue efficiency of h-IL-1R1 in cultured HEK 293 cells. RNAi-resistant h-IL-1R1 was co-transfected with IL-1R1-i1 or with CAG-GFP, respectively. **(D)** Quantitative data are shown as mean ± SEM from three independent experiments. **P <0.01 versus control group. CAG-GFP = pCAG-IRES-EGFP.

Based on the effects of IL-1β on neuronal migration *in vitro*, we further determined whether it plays the same role in the radial migration of neurons during neocortex morphogenesis *in vivo*. Given that a mouse knockout does not show an expected phenotype due to genetic compensation and/or species differences, we employed a method of *in utero* RNAi to knock down IL-1R1 protein levels in migrating neocortical neurons in rats [20]. Two effective siRNA sequences (IL-1R1-i1 and -i2) against IL-1R1 mRNA and a scramble siRNA sequence were designed and cloned into the pSuper vector. The knockdown efficiency of these siRNAs was verified by western blotting in cultured cortical neurons. As illustrated in Figure 4A-B, IL-1R1-i1 and IL1R1-i2 markedly reduced the expression of IL-1R1 protein, whereas scramble siRNA produced no significant effect. Then, the siRNA constructs were electroporated into cortical progenitors at E16 by using *in utero* electroporation, with co-transfection of a plasmid coding for EYFP to label newborn neurons derived from these progenitors.

**Figure 5:**
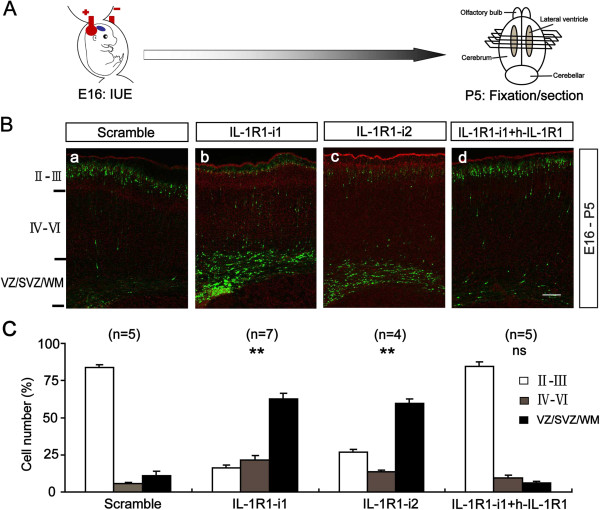
**IL-1R1 RNAi impairs radial migration of cortical neurons. (A)** Schematics of IL-1R1 RNAi procedure. **(B)** Representative images of coronal sections of P5 rat somatosensory cortex transfected at E16 with different siRNAs constructs using IUE including scramble control, two effective siRNA sequences against IL-1R1 (IL-1R1-i1 and -i2) and IL-1R1 siRNA mixed with human IL-1R1 homologue (IL-1R1-i1 + hIL-1R1, rescue). Sections were counterstained with propidium iodide (red). **(C)** Histograms showing the percentages of transfected cells at different regions of P5 rat cortex. Numbers in brackets are numbers of brains analyzed in each group. Scale bar, 100 μm. **P <0.01 versus scramble group. E, embryonic day; IUE, *in utero* electroporation; P, postnatal day.

**Figure 6:**
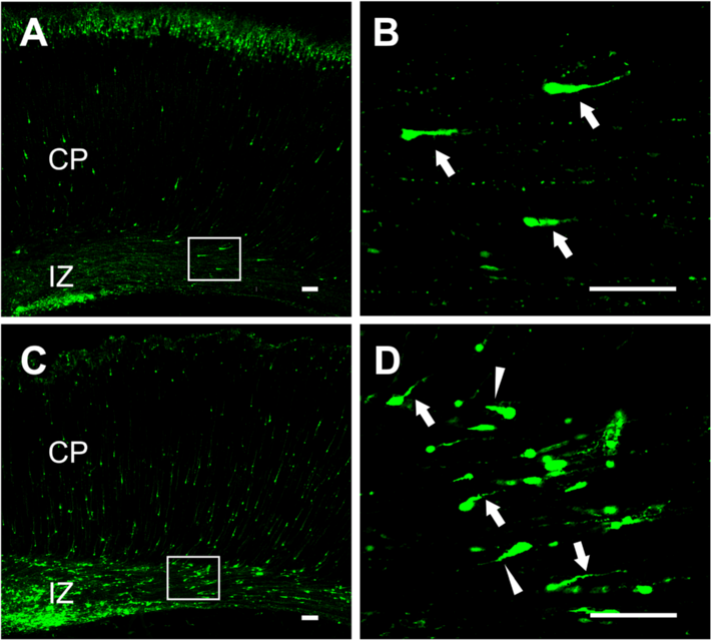
**Misorientation of leading processes of migrating neurons by IL-1R1 down-regulation.** Representative images of migrating neurons from P5 cortices electroporated at E16 with control **(A)** or IL-1R1 siRNA **(C)**. The selected regions in the left panels are shown in high magnification at right **(B** and **D**, respectively). Arrows and arrow heads in **B** and **D** show migrating neurons with normal or reversed leading processes, respectively. Scale bar, 300 μm in A and C, 100 μm in **B** and **D**. E, embryonic day; P, postnatal day.

We examined the position and distribution of transfected cells in the somatosensory cortex at P5 (Figure 5A). In the cortex transfected with scramble plasmid (control), most EYFP-labeled cells (83.60 ± 2.17%) migrated out of the subventricular zone (SVZ) and reached the proper layers (layers II and III) (Figure 5B_a_ and C) as previously reported [16,21,22]. In contrast, neurons transfected with IL-1R1-i1 displayed a severe retardation in radial migration, with about 83% of total cells unable to arrive at the upper cortical layers (layers II and III) (Figure 5B_b_ and C). Most ectopic cells (62.53 ± 4.27%) did not enter the cortical plate and stayed below the white matter. A similar perturbation in radial migration was observed when another effective siRNA of IL-1R1 (IL-1R1-i2) was used for co-transfection (Figure 5B_c_ and C).Notably, IL-1R1 knockdown (IL-1R1-KD) severely affected the morphology of migrating neurons. At P5, control cells (transfected with EYFP alone) in the intermediate zone showed the morphology typical of migrating neurons, with one leading process extending toward the cortical plate. By contrast, many cells expressing IL-1R1-RNAi1 at this location showed misoriented and, even inverted, leading processes (Figure 6). This abnormal orientation of migrating neurons in P5 brains may be explained by a gain of repulsion from the cortical plate after IL-1R1-KD, or a lack of directionality.

**Figure 7:**
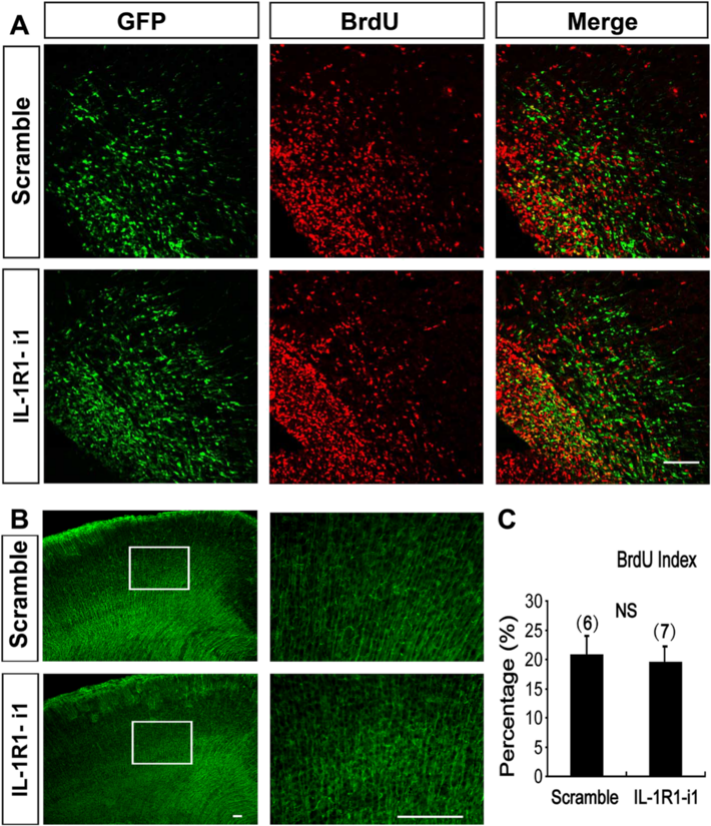
**Down-regulation of IL-1R1 did not affect the proliferation and radial glia scaffold of neural progenitor cells. (A)** Representative images showing BrdU incorporation in control (scramble sequence) and IL-1R1-i1-transfected brains. BrdU was injected intraperitoneally into pregnant rats 24 hours after IUE, and embryos were harvested after another 24 hours. Brain sections were stained with anti-BrdU (red) and anti-GFP (green) antibodies. **(B)** Images of radial glia scaffold counterstained with anti-Nestin antibody in P0 cortices electroporated with scramble or IL-1R1-i1. The selected regions in the left panels are shown in high magnification at right. Transfection with IL-1R1-i1 did not cause obvious changes in the morphology of radial glial scaffold in cortices. **(**C**)** Histograms showing the percentage of BrdU^+^/GFP + cells in total transfected cells (GFP+) (BrdU index) in the two groups. Numbers in brackets are numbers of brains analyzed in each group. No significant differences between scramble and IL-1R1-i1 groups were found (*P* = 0.22, Student’s *t* test). Scale bar, 100 μm. BrdU, bromodeoxuridine; P, postnatal day.

To further confirm the specificity of IL-1R1-mediated neuronal migration, we constructed a RNAi-resistant human IL-1R1 (h-IL1R1) expression vector and verified its efficiency in cultured cortical neurons and human embryonic kidney 293 (HEK 293) cells, as illustrated in Figure 4. Then, we delivered it along with IL-1R1-i1 into the ventricular zone cells and postmitotic migrating neurons of embryonic neocortices at E16. We observed that the rescue of IL-1R1 expression prevented the above-described migration defects, with most transfected neurons (84.52 ± 3.24%) migrating to the appropriate layer (Figure 5B_d_ and C). This indicates that the migration retardation is caused by specific loss-of-function of IL-1R1.Next, we asked whether IL-1R1-KD influences other aspects of cortical development. We examined the effect of IL-1R1-KD on progenitor cell proliferation in the SVZ. We injected BrdU intraperitoneally into pregnant rats 24 hours after IUE and analyzed the BrdU incorporation rate after another 24 hours. We defined a BrdU index as the percentage of BrdU+/GFP + in total transfected cells (GFP+). The result showed no significant difference in BrdU index between the control group (scramble siRNA, 21 ± 1.70%) and the IL-1R1-i1 group (19 ± 2.74%) (Figure 7A and C), suggesting that the proliferation of cortical progenitor cells is not affected after IL-1R1-KD, and that the defective neuronal positioning triggered by IL-1R1-KD does not seem to be the secondary effect of altered proliferation of transfected progenitor cells. We also found that IL-1R1-KD did not cause detectable morphology changes in the radial glial scaffold, along which newborn neurons migrated, as illustrated in Figure 7B.

## Discussion

It is well established that the proinflammatory cytokine IL-1β is a key mediator of inflammation and stress in the CNS [23]. Here, we demonstrate a novel function of IL-1β in brain development: (1) IL-1β is able to attract neuronal migration and growth cone turning *in vitro*; and (2) IL-1R1-KD impairs radial migration of cortical neurons during brain development. These findings suggest that IL-1β signaling contributes to cortical development by guiding neuronal migration.

Previous studies have provided a clue that inflammatory cytokines might play a role in cell migration. Thus, acute brain lesions with an inflammatory element *in vivo* induced a reactive migration of SVZ precursor cells toward the lesion site [24,25]. Similarly, the inflammatory process in the brain attracted the migration of transplanted neural precursor cells (NPCs) into the brain and spinal cord white matter [26]. Our findings further clarify that cortical neurons express IL-1R1 and that the inflammatory cytokine IL-1β can guide the direction of neuronal migration. A recent *in vitro* study showed that IL-1β inhibited the proliferation of neural progenitor cells derived from the E16 rat brain through activation of the SAPK/JNK pathway by using a [^3^H] thymidine incorporation assay [27]. We used *in utero* electroporation to show that knocking down the expression of IL-1R1 in cortical progenitor cells did not significantly affect the proliferation of progenitor cells during development, although other *in vivo* studies revealed that there was an increased proliferation in subventricular adult progenitor cells after various brain injuries that contained inflammatory components [28,29]. The reason for the apparent discrepancy of the developing brain between *in vivo* and *in vitro* studies is currently unknown.

Genetic studies in humans and mice have identified many molecules, deficiencies of which cause defects in neuronal migration [30]. The X-linked dysgenesis that manifests as lissencephaly in males and subcortical heterotopia in females [31,32] has been shown to be due to mutations in the *doublecortin* gene that is highly expressed in fetal brain. Paradoxically, genetic deletion of *doublecortin* gene in mice does not cause neocortical malformation. Similarly, Semaphorin-3A and Robo4 have been demonstrated to guide radial migration of cortical neurons during development [14,33], but Semaphorin-3A -null mice or Robo4 conditional knockout mice do not show obvious defects in cortical layering. These observations may be attributed to the compensatory effects of other guidance factors that play redundant functions during development. Recently, *in utero* RNAi has been introduced as an important addition to traditional mouse knockout studies for studying loss-of–function effects during brain development [34]. Using *in utero* electroporation to knock down the expression of IL-1R1 in cortical progenitor cells, we observed disrupted migration of developing cortical neurons, although IL-1R1 null mice are of normal vigor and display no overt phenotypes or behavioral abnormalities [35]. We further found that co-transfection with h-IL1R1 effectively rescued the down-regulation of IL-1R1 in HEK 293 cells and prevented the migration defects of cortical neurons induced by siRNA. However, we did not verify the overexpression of h-IL1R1 in the triple-transfected GFP-positive cortical neurons by immunofluorescence with anti-IL-1R1 antibodies, which may be a weakness of this study.

Current molecular and genetic studies have greatly expanded our knowledge of brain ontogenesis as well as the genetic mechanism of some neurodevelopment diseases, such as malformations of cortical development (MCD) [36], but it remains unclear how the epigenetic factors, such as *in utero* irradiation, infections, trauma and vascular-ischemic events, cause MCD. The prominent histopathologic features in the brain of patients with MCD include the loss of cortical lamination and neuronal heterotopias [37], suggesting that an aberrant migration of cortical neurons is an essential pathogenetic element. The present study might provide a novel mechanistic clue between IL-1β signaling and MCD. Since IL-1β upregulation is part of a patterned response that unfolds after a wide range of insults including infection, trauma, depression and stroke [4], it would become a noticeable target for the prevention of MCD during pregnancy.

## Conclusions

We first report a novel function of IL-1β during brain development, that is, guiding the neuronal migration, which could have significant implications for the prevention of some neurodevelopment disorders due to abnormal neuronal migration.

## Abbreviations

BDNF: brain-derived neurotrophic factor; BrdU: bromodeoxyuridine; CNS: central nervous system; E: embryonic day; GFP: green fluorescent protein; IL-1β: interleukin-1beta; IL-1RA: IL-1 receptor antagonist; IUE: *in utero* electroporation; MCD: malformations of cortical development; NPCs: neural precursor cells; PBS: phosphate-buffered saline; PCR: polymerase chain reaction; RT: reverse transcriptase; SEM: standard error of the mean; siRNA: small interfering RNA; SVZ: subventricular zone.

## Competing interests

The authors declare that they have no competing interests.

## Authors’ contributions

LM and XWL performed *in utero* electroporation experiments, analyzed the data and contributed to the drafting of the manuscript. SJZ performed transwell and growth cone turning assays. FY and GMZ performed western blotting and immunocytochemistry experiments, and analyzed the data. XBY designed the experiments and analyzed data. WJ conceived and designed the experiments, analyzed data and wrote the manuscript. All authors participated in the critical revision of the manuscript for important intellectual content. All authors read and approved the final manuscript.
